# The lampricide 3-trifluoromethyl-4-nitrophenol causes temporary metabolic disturbances in juvenile lake sturgeon (*Acipenser fulvescens*): implications for sea lamprey control and fish conservation

**DOI:** 10.1093/conphys/coab069

**Published:** 2021-09-08

**Authors:** R Adrian Ionescu, Scott L J Hepditch, Michael P Wilkie

**Affiliations:** 1 Department of Biology and Laurier Institute for Water Science, Wilfrid Laurier University, Waterloo, Ontario N2L 3C5, Canada; 2 Current Address: Centre Eau Terre Environment, Institut National de la Recherche Scientifique, Québec, Québec City G1K 9A9, Canada

**Keywords:** Aquatic invasive speciesfish toxicologyfisheries managementpesticidepiscicidespecies at risk

## Abstract

The pesticide 3-trifluoromethyl-4-nitrophenol (TFM) is applied to rivers and streams draining into the Laurentian Great Lakes to control populations of invasive sea lamprey (*Petromyzon marinus*), which are ongoing threats to fisheries during the lamprey’s hematophagous, parasitic juvenile life stage. While TFM targets larval sea lamprey during treatments, threatened populations of juvenile lake sturgeon (*Acipenser fulvescens*), particularly young-of-the-year (<100 mm in length), may be adversely affected by TFM when their habitats overlap with larval sea lamprey. Exposure to TFM causes marked reductions in tissue glycogen and high energy phosphagens in lamprey and rainbow trout (*Oncorhynchus mykiss*) by interfering with oxidative ATP production in the mitochondria. To test that environmentally relevant concentrations of TFM would similarly affect juvenile lake sturgeon, we exposed them to the larval sea lamprey minimum lethal concentration (9-h LC_99.9_), which mimicked concentrations of a typical lampricide application and quantified energy stores and metabolites in the carcass, liver and brain. Exposure to TFM reduced brain ATP, PCr and glycogen by 50–60%, while lactate increased by 45–50% at 6 and 9 h. A rapid and sustained depletion of liver glucose and glycogen of more than 50% was also observed, whereas the respective concentrations of ATP and glycogen were reduced by 60% and 80% after 9 h, along with higher lactate and a slight metabolic acidosis (~0.1 pH unit). We conclude that exposure to environmentally relevant concentrations of TFM causes metabolic disturbances in lake sturgeon that can lead to impaired physiological performance and, in some cases, mortality. Our observations support practices such as delaying TFM treatments to late summer/fall or using alternative TFM application strategies to mitigate non-target effects in waters where lake sturgeon are present. These actions would help to conserve this historically and culturally significant species in the Great Lakes.

## INTRODUCTION

In the early 20th century, parasitic sea lamprey (*Petromyzon marinus*) invaded the Laurentian Great Lakes, contributing to the devastation of commercial, recreational and culturally important fisheries, along with negative economic impacts on the communities that relied on the lakes and fishery for their livelihoods (Smith and Tibbles, 1980; GLFC, 2011). In response to this ecological and economic emergency, the Great Lakes Fisheries Commission (GLFC) was formed in mid 1950s by the Canadian and US governments, with a mandate to develop a comprehensive program to eliminate/control invasive sea lamprey populations in the Great Lakes basin (GLFC, 2011).

Since the early 1960s, the lampricide, 3-trifluoromethyl-4-nitrophenol (TFM) has been used as part of a program to control populations of invasive sea lamprey in the Great Lakes. Applied to nursery streams infested with larval sea lamprey, TFM treatments take place every 2–4 years, eradicating several generations of sea lamprey at once (Smith and Tibbles, 1980; Bills *et al*., 2003; Boogaard *et al*., 2003; McDonald and Kolar, 2007). TFM is applied at 1.2–1.5 times the sea lamprey minimum lethal concentration (MLC) in order to account for dispersal and dilution of TFM and to maintain the appropriate target concentration as the lampricide block moves down the river. When treating longer rivers (e.g. several kilometres), or in rivers with large water discharges, it is necessary to have additional application stations along lower parts of the river to boost the concentration of TFM (Smith and Tibbles, 1980; Bills *et al*., 2003; Boogaard *et al*., 2003; McDonald and Kolar, 2007).

The use of lampricides has helped to reduce sea lamprey populations in the Great Lakes by ~90% compared to their peak numbers in the mid-20th century (Siefkes, 2017). The success of TFM is mainly due to the much greater sensitivity of sea lamprey to TFM compared to many other fishes due to the lower capacity of their liver to detoxify TFM using glucuronidation (Lech, 1974; Lech and Statham, 1975; Kane *et al.*, 1993, 1994), which renders the lampricide more water soluble and easier to excrete via the urine or faeces (Clarke *et al.*, 1991). In contrast, most non-target fishes can tolerate 3–5 times higher TFM concentrations than that required to kill sea lamprey (Applegate and King, 1962; Bills *et al.*, 2003; Wilkie *et al*., 2019), but there is inter-species variability in the sensitivity to TFM in non-target fishes (Applegate and King, 1962; Marking and Olson, 1975; Bills and Leif, 1976; Johnson *et al.*, 1999; Wilkie et al. 2019), with juvenile lake sturgeon (*Acipenser fulvescens*) being one of the most sensitive (Boogaard *et al.*, 2003). Lake sturgeon populations in the Great Lakes experienced massive declines in the past 200 years due to overharvest, pollution, loss of habitat and barriers to their migration (Auer, 2004; Golder and Associates Ltd, 2011), with populations in the Great Lakes–St. Lawrence drainage designated as threatened by Federal and State legislation in Canada and the USA (Hayes and Caroffino, 2012; COSEWIC, 2017). There have also been concerns that lake sturgeon’s sensitivity to TFM, and its vulnerability to sea lamprey parasitism, particularly in the early juvenile stages could undermine efforts to conserve and restore lake sturgeon populations (Patrick *et al.*, 2009; Hayes and Caroffino, 2012; O’Connor *et al.*, 2017; Dobiesz *et al.*, 2018). Several studies have examined the effects of TFM on lake sturgeon survival (Boogaard *et al.*, 2003; O’Connor *et al.*, 2017), physiology and behaviour (Middaugh *et al.*, 2014; Sakamoto *et al.*, 2016; Hepditch *et al.*, 2019) and TFM-detoxification (Bussy *et al.*, 2017a,b), but none has addressed the mechanism of TFM toxicity, acute effects of TFM exposure on metabolic processes in these fish or the potential effects on their survival. Such knowledge could be very helpful in developing strategies of lampricide application that are compatible with ongoing lake sturgeon conservation and restoration efforts in the Great Lakes.

In recent years, a number of studies have described the physiological impact and mechanism of TFM toxicity in sea lamprey, non-target fishes and other organisms (Viant *et al.*, 2001; Wilkie *et al.*, 2007; Birceanu *et al.*, 2009, 2011, 2014; Clifford *et al.*, 2012; Henry *et al.*, 2015). Because of the structural similarity of TFM to 2,4-dinitrophenol (2,4-DNP), a known uncoupler of oxidative phosphorylation, it was first suggested by Applegate *et al.* (1966) that TFM may cause death by interfering with mitochondrial ATP production. In support of this hypothesis, Niblett and Ballantyne (1976) noted that TFM exposure of isolated rat mitochondria increased the ADP/O ratio, which is indicative of lower ATP production due by uncoupling oxidative phosphorylation. Birceanu *et al.* (2009, 2011) later showed that TFM uncouples oxidative phosphorylation in mitochondria isolated from the livers of sea lamprey and in non-target rainbow trout, leading to a mismatch between ATP supply and ATP demand (Birceanu *et al.*, 2009, 2014). Notably, TFM significantly depleted anaerobic energy stores such as glycogen and phosphocreatine in metabolically active tissues such as the brain, liver and kidney, but less so in the muscle (Wilkie *et al.*, 2007; Birceanu *et al.*, 2009, 2014; Clifford *et al.*, 2012).

The goal of this study was to learn more about the physiological effects and mechanism of TFM toxicity in lake sturgeon to better assess how incidental TFM exposure could affect their physiology and survival in the wild. We predicted that the physiological responses to TFM in lake sturgeon would lead to a multi-system depletion of energy stores, similar to those reported for sea lamprey (Wilkie *et al.*, 2007; Birceanu *et al.*, 2009; Clifford *et al.*, 2012) and for rainbow trout (Birceanu *et al.*, 2011, 2014). To test our hypothesis, juvenile lake sturgeon were exposed for 9 h to the previously published sea lamprey 9-h LC_99.9_ of TFM, also referred to as the MLC required to kill 99.9% of sea lamprey over this time frame, based on an established alkalinity and pH treatment model (Bills *et al.*, 2003).

## Method and materials

### Experimental animals and set-up

Young-of-the-year (YOY; total length, 118 ± 1.9 mm; weight, 4.36 ± 0.2 g; *N* = 32) lake sturgeon were kindly provided by Dr Gary Anderson, Department of Biological Sciences, University of Manitoba. They were the progeny of 4 female and 5 male lake sturgeon, caught on the Winnipeg River (50^o^17′52′′N, 95^o^32′51′′W), from which eggs and sperm were collected by members of the Anderson laboratory in April 2015, fertilized and hatched in mid–late May 2015 (Bjornson and Anderson, 2018). The YOY lake sturgeon were subsequently shipped to Wilfrid Laurier University on 5 December 2015, where they were housed in a multi tank G-HAB aquatic system (Pentair Aquatic Eco-Systems, Apopka, FL, USA) equipped with a five-phase filtration (large particulate, biological, small particulate, carbon and UV) system and kept on recirculation with partial water replacement of 120 l per day. Water was a 50:50 mix of dechlorinated City of Waterloo tap water and reverse osmosis (RO) water resulting in the following water chemistry: pH 8.0, alkalinity of ~200 mg l^−1^ as CaCO_3_, temperature of 14–15°C, dissolved O_2_ (DO) ≥ 90%. Water quality parameters (temperature, pH, dissolved oxygen and conductivity as a proxy for alkalinity) were checked daily, while chlorine and total ammonia were checked weekly. Stocking densities were ~12 g sturgeon biomass l^−1^, and the fish were fed twice daily with frozen blood worms (~2% total body mass; Brine Shrimp Direct, Ogden, UT, USA). Holding and experiments followed Canadian Council of Animal Care guidelines and were approved by the Wilfrid Laurier University Animal Care Committee.

Due to the unusually high hardness and alkalinity of City of Waterloo tap water, the lake sturgeon were acclimated and exposed to TFM in reconstituted water of lower hardness and alkalinity, 4 weeks prior to experiments. The acclimation set-up comprised two 37-l glass aquaria, both connected to an external 150 -l reservoir (~225 l recirculating system). The system was filled with reconstituted water set to the desired nominal alkalinity of 150 mg l^−1^ as CaCO_3_, at water pH of approximately 8.4, dissolved oxygen concentration >90% saturation and a temperature of 13°C (Table 1). The reconstituted water was prepared using methods described by the APHA (2007). Briefly, RO water was reconstituted with addition of adequate amounts of CaSO_4_•H_2_O (BioShop Canada Inc. Burlington, Ontario, Canada), KCl (VWR International LLC, West Chester, PA, USA) and MgSO_4_ (BioShop Canada Inc.) to control water hardness (~100 mg l^−1^ CaCO_3_) and NaHCO_3_ (BioShop Canada Inc.) to establish desired alkalinity. Each aquarium was fitted with an activated carbon and biological filtration system, with the recirculating system receiving a 50% water change every other day. Water pH, dissolved oxygen, temperature and conductivity were checked daily, and alkalinity was measured following the preparation of each batch of reconstituted water. During acclimation, the fish continued to be fed daily on frozen blood worms (2% of body weight), as described above.

**Table 1:** Mean water alkalinity, pH, dissolved O2 (DO) and temperature in aquaria housing lake sturgeon during the pre-experimental acclimation period and in the experimental (TFM-exposure) water

Aquaria	Alkalinity (as CaCO_3_ L^−1^)	pH	DO%	Temperature (°C)	[TFM] (mg L^−1^)
Acclimation	148 ± 2	8.43 ± 0.02	93.7 ± 0.3	13.6 ± 0.1	N/A
Experimental	150 ± 1	8.36 ± 0.01	93.0 ± 0.3	13.9 ± 0.1	*4.6 ± 0.1*

Measurements were conducted daily during acclimation and water parameters plus TFM were measured throughout the experiments (0, 3, 6, 9 h; *N* = 4 per sample period), expressed as mean ± SEM.

### Experimental protocol

Applications of TFM typically take place over ~12 h and are designed to ensure that the larval sea lamprey in a given reach of river or stream are exposed to at least the MLC required to kill 99.9% of lamprey (the 9-h LC_99.9_) over a minimum exposure period of 9 h (Barber and Steeves, 2019). By exposing the sturgeon to the 9-h MLC of lamprey, our study conditions therefore mimicked those likely to be encountered during an actual treatment in the field. The TFM exposures were conducted using field formulation TFM (Clariant SFC GMBH WERK, Griesheim, Germany; 35% active ingredient dissolved in isopropanol), provided courtesy of the Sea Lamprey Control Centre, Fisheries and Oceans Canada (DFO), Sault Ste. Marie, Ontario.

For each set of experiments, lake sturgeon (*N* = 8 per experimental series; *N* = 32 total) were randomly transferred one at a time to darkened, aerated exposure-chambers (volume, 750 ml; *N* = 1 sturgeon per container) the night before experiments. Each container was continuously fed with aerated water of identical chemistry to the acclimation water described above (flow rate, ~1.0 l min^−1^). The next day, the lake sturgeon were sampled under control conditions (no TFM; N = 8) or following exposure to a nominal concentration of 4.7 mg l^−1^ (measured: 4.6 ± 0.1 mg l^−1^) of TFM, which was equivalent to the sea lamprey MLC, determined from tables published by Bills *et al.* (2003), based on the measured water pH and alkalinity of the test water.

No mortalities were observed during TFM exposure; thus, experimental results were not confounded by variability in survivorship. All experiments were conducted at the same time of day and dosing of the exposure chambers with TFM was staggered by 5 min in order to allow for collection of tissues at the end of exposure. Lake sturgeon were subsequently sampled under control conditions (*N* = 8) or following 3, 6 or 9 h of TFM exposure (*N* = 8 per sample period). The control lake sturgeon not exposed to TFM were sampled at the start (*N* = 4) and at the conclusion (*N* = 4) of experiments to rule out possible temporal variation in physiological parameters (energy stores, metabolites, pH) to be measured. Because no variation was observed, the controls sampled at the start of the experiment were combined (pooled) with those sampled at the end of the experiment. At each sample period, the sturgeon were euthanized with an overdose of 1.0 g l^−1^ tricaine methanesulfonate (Syndel Laboratories Canada, Nanaimo, British Columbia, Canada) buffered with 2.0 g l^−1^ NaHCO_3_. Each fish was then patted dry with paper towel, weighed and measured for total length. The liver and brain were then excised, snap frozen in liquid nitrogen and stored at −80°C in 1.5 ml microcentrifuge tubes for later analyses. The carcass was freeze clamped in aluminium foil using pre-chilled aluminium tongs in liquid nitrogen and stored at −80°C for later analyses (Wang *et al.*, 1994a). Water samples from each tank were collected at the beginning of the experiment and every 3 h thereafter, followed by the immediate measurement of TFM concentration. Water chemistry was measured throughout the experiment and remained relatively stable (Table 1).

### Analytical procedures

#### Water chemistry and TFM concentration

Water TFM concentration was measured using a plate spectrophotometer (absorbance, 395 nm; Epoch2 Microplate Reader, BioTek Winooski, VT, USA) and precision standards (provided courtesy of USGS, Hammond Bay, MI, USA) based on a micro-modification of GLFC Standard Operating Procedure 43.1.0, Hammond Biological Station, Millersburg, Michigan. Water alkalinity was measured using a commercial kit (Hach, Alkalinity Test Kit, Model AL-AP, Hach Canada, Mississauga, Ontario) and water pH using a handheld pH metre (pH 11 metre, Oakton Instruments, IL, USA). Water Ca^2+^ and Mg^2+^ concentrations were measured using flame atomic absorption spectroscopy (AAS, PinAAcle 900 T, Perkin Elmer, Waltham, MA, USA).

#### Tissue processing and metabolite quantification

Tissue metabolite extraction procedures followed those outlined in Bergmeyer (1983) as described in Wilkie *et al*. (1997). Briefly, lake sturgeon carcasses were ground to a fine powder under liquid nitrogen, using an insulated mortar and pestle. Samples were acidified for 10 min with 4 volumes of 8% perchloric acid (PCA) containing 1 mmol l^−1^ ethylenediaminetetraacetic acid (EDTA, Sigma-Aldridge Canada, Oakville, Ontario, Canada), then centrifuged at 4°C and 10 000 *g* for 5 min. An aliquot (sub-sample 1) of supernatant was neutralized (~pH = 7) using 3 mol l^−1^ K_2_CO_3_ (VWR International LLC), and set aside for glucose and glycogen analysis. The remainder of the supernatant (sub-sample 2) used for measurement of ATP, phosphocreatine (PCr), pyruvate and lactate were neutralized (~pH = 7) in a half volume of 2 mol l^−1^ KOH (EDM Millipore Canada Ltd, Etobicoke, Ontario, Canada). After preparation, all the homogenized samples were flash frozen in liquid nitrogen and stored at −80°C until analysed. Rainbow trout white muscle, which was used a positive control for all assays, was extracted and stored in a similar manner. Procedures for analysing brain and liver tissue were similar to carcass/muscle but due to the smaller amounts of tissue available, 4 volumes 8% PCA containing 1 mmol l^−1^ EDTA was added to microcentrifuge tubes containing 100–150 mg of the tissues and homogenized on ice using a hand-held, motorized, plastic pestle homogenizer (Gerresheimer Kimble Kontes LLC, Dusseldorf, Germany), flash frozen in liquid nitrogen and stored at −80°C as described above.

Tissue (carcass, brain and liver) glucose and glycogen was determined using the neutralized extract from sub-sample 1 to which one-part 2 mol l^−1^ acetate buffer was added followed by 40 units (U) of amyloglucosidase (Sigma-Aldridge Canada) to catalyse the conversion of glycogen to glucose during a 2 h incubation at 37°C. The glycogen digestion was terminated by addition of 70% PCA and neutralized using 3 mol l^−1^ K_2_CO_3_. An aliquot of sub-sample 1, to which no amyloglucosidase was added, was set aside and used for the determination of free glucose. The glycogen concentration in the tissue was based on the total glucose concentration measured in the amyloglucosidase treated sample minus the free glucose, yielding the glycogen concentration in μmol glucosyl units g^−1^ wet weight. Tissue free glucose and total glucose content were spectrophotometrically analysed enzymatically (hexokinase; HK) at 340 nm (Epoch 2, BioTek Inc., Burlington, VT, USA).

The neutralized carcass extracts obtained from sub-sample 2 were analysed spectrophotometrically at 340 nm (BioTek, Epoch 2) using micro-modification of enzymatic assays outlined in Bergmeyer (1983) for ATP [HK and glucose-6-phosphatase (G6PDH)], PCr [creatine kinase (CK)], ADP [pyruvate kinase (PK) and lactate dehydrogenase (LDH)], creatine (CK, PK and LDH), pyruvate (LDH) and lactate (LDH). Assays on brain and liver were restricted to ATP, PCr, glycogen and lactate due to the limited amounts of tissue that were available. Energy stores and metabolites, except glycogen, were expressed as μmol g^−1^ wet weight.

The method for measuring intracellular pH (pHi) followed those of Pörtner (1990). Briefly, carcass (trunk minus internal viscera) was ground to a fine powder under liquid nitrogen using an insulated mortar and pestle. Slurries were created by combining ~100 mg of ground tissue with 400 µl ice-cold metabolic inhibitor cocktail containing 150 mmol l^−1^ KF and 6 mmol l^−1^ nitrilotriacedic acid sodium salt (Na_2_NTA). The samples were vortexed for 10 s and pulsed in a centrifuge for ~10 s at 4°C. The resultant supernatant was used to measure pH at 15°C (acclimation temperature of the experimental fish) using a micro pH probe (Hamilton Bonaduz AG, Bonaduz, Switzerland) and metre (ION85 Analyzer, Radiometer, Copenhagen, Denmark). The pH electrode was calibrated using clinical standards (pH 7.0 and pH 10.0; VWR International LLC, Mississauga, Ontario, Canada) prior to measurement of samples and checked for drift several times during the measurement process. All pH readings of samples and pH standards were allowed to stabilize for 3 min before final readings were recorded.

#### Statistical analyses

Data were analysed using one-way analysis of variance (ANOVA), followed by Tukey’s multiple comparison test. In instances where the data were not normally distributed, or there was inequality of variances, data was analysed using non-parametric ANOVA (Kruskal–Wallis test), followed by Dunn’s multiple comparison test. All data were expressed as the mean ± SEM, with the level of significance set to *P* ≤ 0.05. All statistical data analyses were performed using Prism® 7.03 (GraphPad Software Inc, La Jolla, CA, USA).

## Results

### Effects of TFM on energy stores and metabolites in lake sturgeon brain

**Figure 1:**
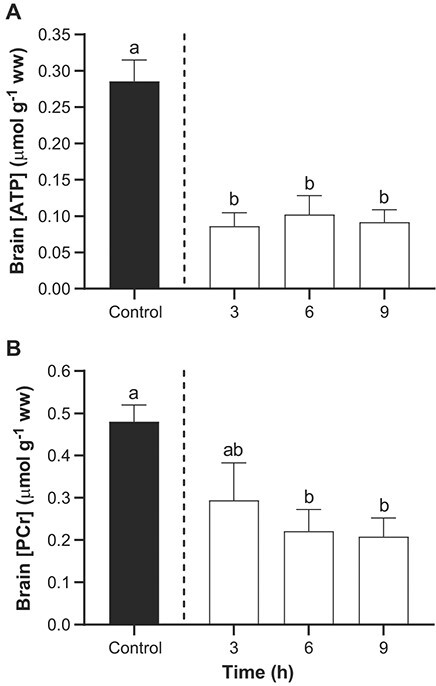
Effects of TFM exposure on ATP and phosphocreatine in the brain of lake sturgeon. Changes the concentration of (A) adenosine triphosphate (ATP) and (B) phosphocreatine (PCr) in the brains of lake sturgeon under control conditions (non-exposed; solid bar) and during exposure to TFM at a measured concentration of 4.6 ± 0.1 mg l^−1^ for 3, 6 and 9 h (open bars). Data are expressed as the mean ± S.E.M. (*n* = 8). Different lowercase letters indicate significant differences between each treatment group and controls (*P* ≤ 0.05).

The concentration of ATP in the brain of control lake sturgeon, not exposed to TFM, averaged 0.29 ± 0.03 μmol g^−1^ ww and phosphocreatine averaged 0.48 ± 0.04 μmol g^−1^ ww. Following exposure to TFM, the concentration of ATP in the brain underwent an immediate and sustained reduction of ~60% compared to the control measurements and PCr was reduced by ~50% at 6 and 9 h (Fig. 1).

**Figure 2:**
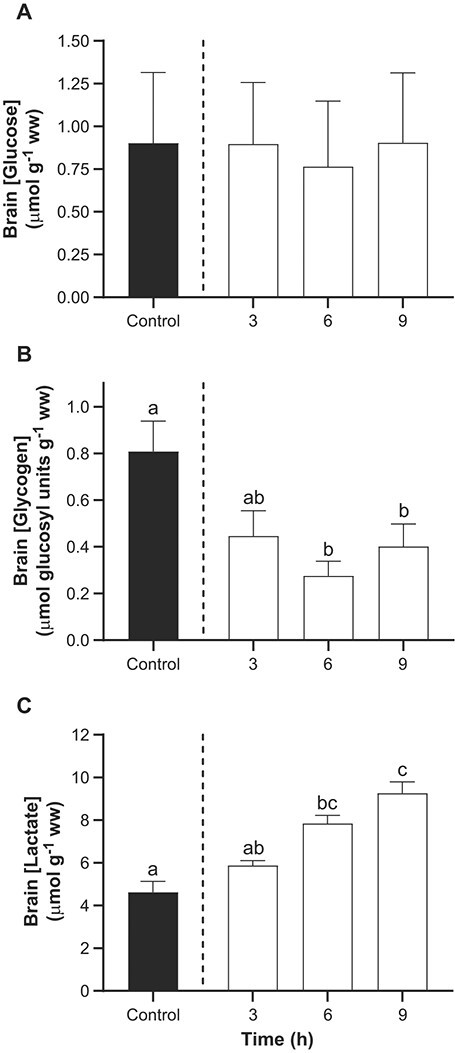
Effects of TFM exposure on glucose, glycogen and lactate in the brain. Changes in the concentrations of (A) glucose, (B) glycogen and (C) lactate in the brains of lake sturgeon under control conditions (non-exposed; solid bar) and during exposure to TFM at a measured concentration of 4.6 ± 0.1 mg l^−1^ for 3, 6 and 9 h (open bars). Data are expressed as the mean ± S.E.M. (*n* = 7–8). Different lowercase letters indicate significant differences between each treatment group and controls (*P* ≤ 0.05).

Brain glucose and glycogen concentrations were relatively low in lake sturgeon, averaging 0.81 ± 0.13 μmol g^−1^ ww and 0.13 μmol g^−1^ ww, respectively. Brain glucose concentrations were unchanged in the presence of TFM (Fig. 2A) but glycogen was significantly depleted by 50% at 6 and 9 h relative to the control fish (Fig. 2B). Lactate levels in TFM exposed sturgeon brain significantly increased from 4.6 ± 0.5 μmol g^−1^ ww in the controls to 7.9 ± 0.4 μmol g^−1^ ww after 6 h, peaking at 9.3 ± 0.5 μmol g^−1^ ww after 9 h, an increase of approximately 2-fold (Fig. 2C).

### Effects of TFM on liver glucose and glycogen reserves

**Figure 3:**
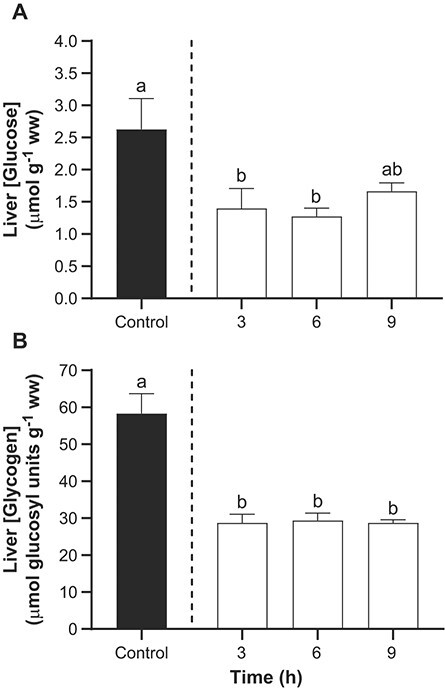
Effects of TFM exposure on hepatic glucose and glycogen reserves. Changes in the concentrations of (A) glucose and (B) glycogen in the liver of lake sturgeon under control conditions (non-exposed; solid bar) and during exposure to TFM at a measured concentration of 4.6 ± 0.1 mg l^−1^ for 3, 6 and 9 h (open bars). Data are expressed as the mean ± S.E.M. (*n* = 8). Different lowercase letters indicate significant differences between each treatment group and controls (*P* ≤ 0.05).

Liver glucose and glycogen concentrations were markedly depleted in lake sturgeon exposed to TFM. In control animals, liver glucose concentrations averaged 2.6 ± 0.5 μmol g^−1^ ww, while liver glycogen concentrations were much higher averaging 58.3 ± 5.4 μmol glucosyl units g^−1^ ww (Fig. 3). Following exposure to TFM, liver glucose concentrations were ~50% lower than controls at 3 and 6 h, but not quite significantly different at 9 h (*P* = 0.11; Fig. 3A). In contrast, liver glycogen concentrations were significantly reduced by ~50% at all time points (Fig. 3B).

### Effects of TFM on energy stores, metabolites and pHi in carcass

**Figure 4:**
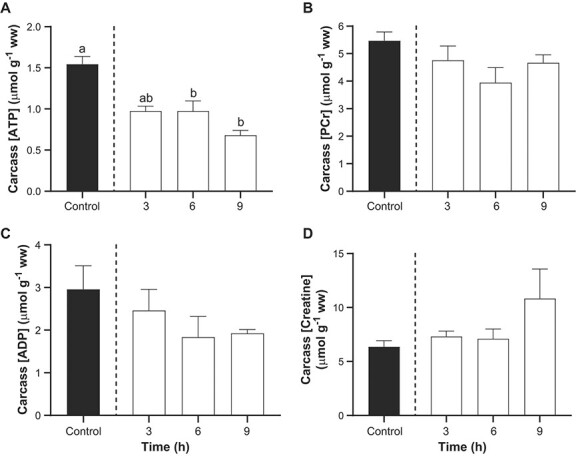
Effect of TFM exposure on ATP and phosphocreatine in lake sturgeon carcass. Changes in the concentrations of (A) adenosine triphosphate (ATP), (B) Phosphocreatine (PCr), (C) adenosine diphosphate (ADP) and (D) creatine in the carcass of juvenile lake sturgeon under control conditions (non-exposed; solid bar) and during exposure to TFM at a measured concentration of 4.6 ± 0.1 mg l^−1^ for 3, 6 and 9 h (open bars). Data are expressed as the mean ± S.E.M. (n = 8). Different lowercase letters indicate significant differences between each treatment group and controls (*P* ≤ 0.05).

The carcass comprised the trunk (including the caudal fins) of the sturgeon, minus the liver, kidney and other viscera. In the controls, sturgeon carcass ATP and PCr concentrations averaged 1.5 ± 0.1 μmol g^−1^ ww and 5.5 ± 0.3 μmol g^−1^ ww, respectively (Fig. 4). However, in the group exposed to TFM, there was a marked reduction in ATP concentrations of ~56% by 9 h of exposure compared to the control fish (Fig. 4A). However, there were no notable changes in PCr (Fig. 4B), ADP (Fig. 4C) and creatine (Fig 4D).

**Figure 5:**
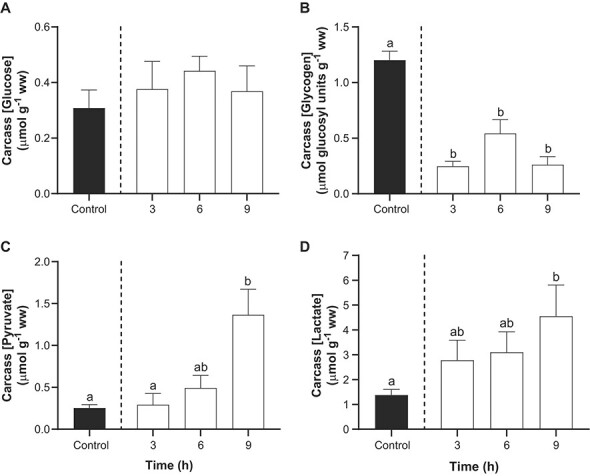
Effects of TFM exposure on glucose, glycogen and related metabolites in the carcass of lake sturgeon. Changes in carcass concentrations of (A) glucose, (B) glycogen, (C) pyruvate and (D) lactate in the carcass of juvenile lake sturgeon under control conditions (non-exposed; solid bar) and during exposure to TFM at a measured concentration of 4.6 ± 0.1 mg l^−1^ for 3, 6 and 9 h (open bars). Data are expressed as the mean ± S.E.M. (*n* = 8). Different lowercase letters indicate significant differences between each treatment group and controls (*P* ≤ 0.05).

**Figure 6:**
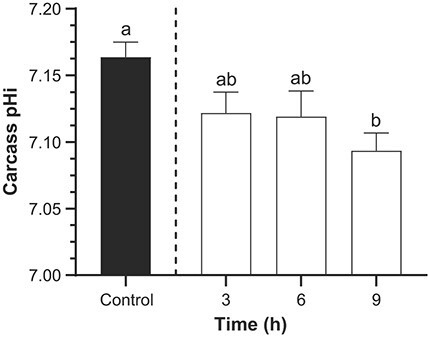
Effect of TFM exposure on intracellular pH in the carcass juvenile lake sturgeon. Changes in pHi in the carcass of juvenile lake sturgeon under control conditions (non-exposed; solid bar) and during exposure to TFM at a measured concentration of 4.6 ± mg 0.1 l^−1^ for 3, 6 and 9 h (open bars). Data are expressed as the mean ± S.E.M. (*n* = 8). Different lowercase letters indicate significant differences between each treatment group and controls (*P* ≤ 0.05).

Glucose concentrations in the carcass of TFM-exposed lake sturgeon did not significantly differ from controls (Fig. 5A), but glycogen concentrations were significantly affected, declining by ~50–80% compared to the control values of 1.20 ± 0.08 μmol glucosyl units g^−1^ ww (Fig. 5B). Concomitantly, there were 5-fold and 3.5-fold increases in carcass pyruvate (Fig. 5C) and lactate levels (Fig. 5D), respectively, at 9 h of TFM exposure. Despite the marked declines in glycogen and increased lactate, TFM exposure only resulted in a mild acidosis in the carcass in which intracellular pH (pHi) dropped from 7.16 ± 0.01 in non-exposed control sturgeon, to pH 7.09 ± 0.01 following 9 h of TFM exposure (Fig. 6).

## Discussion

### TFM interferes with ATP production and glucose homeostasis in lake sturgeon

Exposure of juvenile lake sturgeon to TFM, at concentrations that could be encountered during a typical field application of the lampricide, resulted in significant decreases in brain ATP, PCr and glycogen, with corresponding increases in lactate. These observations are consistent with TFM’s established mode of action, in which it disrupts mitochondrial ATP production by interfering with oxidative phosphorylation (Niblett and Ballantyne, 1976; Birceanu *et al.*, 2011), increasing reliance on anaerobic metabolic processes, such as the dephosphorylation of phosphocreatine to buffer ATP supplies, and glycolysis (Wilkie *et al.*, 2007; Birceanu *et al.*, 2009, 2014; Clifford *et al.*, 2012). As noted previously, both the supply of PCr and glycogen, the primary substrate of glycolysis, are finite, and when these reserves are lowered, and ATP supply can no longer match ATP demands, the fish are unable to maintain homeostasis, leading to death (Wilkie *et al.*, 2019).

A key target organ of TFM is the brain, one of the most metabolically active organs in vertebrates (Magistretti, 1999), which accounts for 2.7–3.4% of total body energy expenditure in ectotherms (Sokoloff, 1989; Hylland *et al.*, 1997; Purdon and Rapoport, 1998). The ATP supply to the brain is sustained by the oxidation of glucose, which is consumed at a higher rate per unit mass than any other organ in rainbow trout (Washburn *et al.*, 1992). Glucose is primarily supplied via the plasma, mainly through the catabolism of endogenous glycogen stores in the liver, but also supplied by the brain itself in some cases (Soengas and Aldegunde, 2002; Polakof *et al.*, 2007, 2012). Glycogen stores are relatively low in the brain of the lake sturgeon, especially in relation to larval sea lamprey in which brain glycogen can exceed 100 μmol glucosyl units g^−1^ ww (Rovainen, 1970; Foster *et al.*, 1993; Clifford *et al.*, 2012), and in fishes that are hypoxia or anoxia tolerant such as the crucian carp (*Carassius carassius*), in which glycogen concentrations can reach 200 μmol glucosyl units g^−1^ ww (Vornanen and Paajanen, 2006). High brain glycogen in these fishes likely serves as an important anaerobic energy reserve when O_2_ supply to the brain is limited, allowing the fish to rely on glycolysis to sustain ATP production when supply is limited due to impaired or absent aerobic metabolism (Rovainen, 1970; Vornanen and Paajanen, 2006). Other less hypoxia-tolerant fishes, including the lake sturgeon, primarily rely on liver glycogen reserves to meet the glucose demands of the nervous system, with rates of glycogenolysis increasing in response to glucagon release as glucose supply diminishes (Polakof *et al.*, 2012).

In most vertebrates an inability of the brain to oxidize glucose due to a lack of oxygen (ischemia, environmental anoxia or hypoxia) or compounds that directly interfere with oxidative phosphorylation such as TFM (Niblett and Ballantyne, 1976; Birceanu *et al.*, 2011; Huerta *et al.*, 2020) would obviously impair brain function by restricting its ATP supply (Soengas and Aldegunde, 2002; Polakof *et al.*, 2012). While ATP demands might be temporarily met through greater reliance on anaerobic glycolysis, this process also results in the generation of lactate and metabolic acid (H^+^), which could result in corresponding decreases in intracellular pH further compromising neural function. Due to limited tissue availability, we did not measure brain pHi in the present study. However, we postulate that brain pHi was likely reduced due to the generation of metabolic acidosis arising from increased reliance on anaerobic glycolysis. At first glance, this prediction appears to contradict studies on the white sturgeon (*Acipenser transmontanus*), which preferentially control intracellular pH in tissues such as brain, muscle, heart and liver when faced with decreases in extracellular (plasma) pH arising from respiratory acid–base disturbances such as hypercarbia (elevated external P_CO2_) or hyperoxia (Brauner and Baker, 2009; Shartau *et al.*, 2016). However, when white sturgeon experienced a metabolic acidosis due to transient (5 min) exposure to anoxia, preferential pHi regulation was absent in tissues including the brain, liver and white muscle (Shartau *et al.*, 2017). Based on these observations, and the depletion of glycogen and the marked build-up of lactate we observed, we suggest that a decrease in brain pHi was likely, but the extent of the disturbance will not be known until these measurements are made under similar conditions. Further support for this hypothesis was observed in the muscle, where we did observe a slight, but significant metabolic acidosis, which was accompanied by pronounced declines in glycogen and increased lactate during TFM exposure (Fig. 6; see below).

In some respects, the present findings in juvenile lake sturgeon are similar to observations made in larval sea lamprey, where it was reported that exposure to sufficiently high concentrations of TFM results in severe depletion of glycogen reserves in the brain (Clifford *et al.*, 2012). However, the very low basal (control) concentrations of glycogen in the brain of the lake sturgeon, compared to the larval sea lamprey, suggests that the response was of lesser consequence in the lake sturgeon, which likely relied in exogenous sources of glucose such as the liver (see below). Ectothermic vertebrates, such as Agnathans and benthic fishes, which may experience frequent periods of hypoglycemia due to factors such as non-trophic life stages (during metamorphosis) and/or limited food supply (e.g. during winter), tend to depend on endogenous brain glycogen as a proximate energy source, rather than exogenous carbohydrates; however, these fishes generally have very high levels of brain glycogen (Plisetskaya, 1985; Schmidt and Wegener, 1988; Foster *et al.*, 1993; Henry *et al.*, 2015). The relatively low concentrations of brain glycogen observed in the present study suggest that this is not the case in the brain of lake sturgeon. Instead, increased rates of glycogenolysis in the liver, which stores approximately 60-fold higher glycogen concetrations than the brain, would sustain glucose supply to the circulatory system and brain, as observed in other aquatic and terretrial vertebrates (Soengas and Aldegunde, 2002; Polakof *et al.*, 2012). The fact that lactate concentrations were 10-fold higher than the observed decreases in brain glycogen strongly suggests that there was increased anaerobic metabolism of circulatory glucose, rather than endogenous brain glycogen, during TFM exposure.

As previously demonstrated, oxidative ATP production in the mitochondria is impeded by TFM, triggering rapid depletion of energy stores, not only in the brain, but in other vital, metabolically active tissues such as the liver (Birceanu *et al.*, 2009, 2014). Indeed, glycogen levels in the liver were severely depleted and remained depleted for the duration of the present experiments. Since liver glycogen is critical for the maintenance of glucose homeostasis in the circulation and nervous system of vertebrates (Panserat *et al.*, 2000), it is likely that the severe glucose and glycogen depletion observed in the liver of TFM-exposed lake sturgeon reflected the mobilization of glycogen stores in response to reduced glucose supply to the brain. Ultimately, when the liver glucose and glycogen stores were exhausted due to TFM exposure, the brain would be starved of glucose, its primary fuel source, leading to death (Wilkie *et al.*, 2007).

### Potential impact of TFM on ecological and physiological performance by lake sturgeon

In conjunction with impairment of central nervous system function, TFM could also adversely affect physiological processes that impact lake sturgeon survival. For instance, the diminished energy stores (ATP, PCr and glycogen) observed in the central nervous system (brain) of TFM exposed lake sturgeon could have also occurred in the peripheral nervous system, where olfactory sensory neuron function could have also been impaired. Indeed, the mechanisms of olfactory impairment could be at the level of the olfactory neurons themselves, where ATP would be needed to repolarize neurons following action potentials. Presumably, TFM’s inhibition of oxidative phosphorylation would extend to olfactory neurons, which are also enriched with mitochondria (Zielinski *et al.*, 1996; Daghfous *et al.*, 2012; Fluegge *et al.*, 2012). A recent study on YOY lake sturgeon using electro-olfactography, which measures the electrical activity of olfactory sensory neurons (OSNs) in response to odorants, demonstrated that exposure to environmentally relevant concentrations of TFM significantly impaired olfaction (Sakamoto *et al.*, 2016). The impaired electrical responses of both microvillous OSNs, which are involved in detecting food cues, and ciliated OSNs involved in detecting migration and alarm cues, suggested that both feeding and predator detection could be impaired (Sakamoto *et al.*, 2016). Accompanying behavioural studies demonstrated that TFM-exposed fish were in fact less able to detect food cues in the water, which was accompanied by reduced food consumption, suggesting that TFM exposure could potentially compromise growth. However, such compromise may only be temporary, as suggested by the absence of short-term growth (14 d) observed in TFM-exposed (2.5, 5.0, 7.5 mg l^−1^) fingerling lake sturgeon (~146 mm in length) for 12 h (Middaugh *et al.*, 2014). Predator avoidance was not tested, but an earlier mesocosm study demonstrated that TFM-exposed fathead minnow (*Pimephales promelas*) did not show diminished predator avoidance of largemouth bass (*Micropterus dolomieu*; Middaugh *et al.*, 2014).

Aside from physiological disturbances arising from TFM exposure in the nervous system and liver, profound metabolic disturbances were also observed in the carcass, which would mainly reflect events occurring in the muscle because the head and viscera had been removed prior to analysis. Previous studies have demonstrated that there is significant accumulation of TFM in the white muscle of fishes during lampricide exposure, which is normally eliminated within 24 h (Vue *et al.*, 2002; Hubert *et al.*, 2005; Foubister, 2018). The white muscle would therefore have been vulnerable to TFM-induced impairment of oxidative phosphorylation, as reported previously for larval sea lamprey and rainbow trout exposed to their respective TFM 12-h LC_50_’s, in which there were marked declines in glycogen and increased lactate (Birceanu *et al.*, 2009, 2014). These observations, plus the decrease in muscle intracellular pH, are consistent with increased reliance on glycolysis in the tissue. It seems less likely that the muscle contributed to glucose homeostasis in the body during TFM exposure, given the apparent dominance of the liver in maintaining glucose homeostasis in lake sturgeon (see above) and the very low resting concentrations of glycogen to begin with in the carcass/muscle of lake sturgeon. In fact, these low basal glycogen concentrations of glycogen were below respective measurements reported in white sturgeon and shortnose sturgeon (*Acipenser brevirostrum*), which were approximately 10-fold higher (Kieffer *et al.*, 2001; Patterson *et al.*, 2017). These differences are likely because the fingerling sturgeon we used were much younger and smaller (~4.4 g; see above) than the white sturgeon (~124 g; Patterson *et al.*, 2017) and shortnose sturgeon (18–20 g; Kieffer *et al.*, 2001), but interspecies variability might also explain some of the differences.

Whether or not the decline in muscle glycogen would negatively impact physiological performance in sturgeon remains unresolved. In many fishes white muscle glycogen fuels burst swimming (Kieffer, 2000), which in salmonid fishes results in profound decreases in intramuscular glycogen with corresponding increases in lactate (Milligan and Wood, 1986; Schulte *et al.*, 1992; Wang *et al.*, 1994b; Wilkie *et al.*, 1997). Surprisingly, few studies have examined exercise physiology in lake sturgeon. However, Kieffer *et al.* (2001) demonstrated that the physiological disturbances arising from exercise in shortnose sturgeon were much less than in other fishes, particularly salmonids, as characterized by much lower rates of excess post-exercise oxygen consumption, which was likely related to the lower amounts of lactate accumulation, less pronounced reductions in PCr and minimal changes in intramuscular glycogen concentration that were observed (Kieffer *et al.*, 2001). While less pronounced changes in PCr and glycogen were observed in shortnose sturgeon than salmonid fishes, the respective increases in pyruvate and lactate, 2.5-fold and 7.0-fold, suggests that these also relied on glycolysis during periods of short-term intensive exercise, which would be required to evade predators. Thus, the near depletion in carcass (muscle) glycogen stores that was observed in the lake sturgeon during TFM exposure could potentially impair burst swim performance capacity. Indeed, video recordings suggested that there were reductions in maximum swimming velocity and acceleration in juvenile lake sturgeon exposed to the MLC of TFM (Sakamoto *et al.*, 2016). It would be most informative to follow-up these intriguing findings using swim performance tests designed to determine how TFM exposure, not to mention the critical post-exposure recovery phase, affects acceleration and burst and prolonged (exhaustive) swimming performance in lake sturgeon and other non-target fishes.

### Implications for lake sturgeon conservation and sea lamprey control

Sea lamprey parasitism of culturally significant, commercial and recreational fishes still has the potential to cause serious harm to Great Lake’s fisheries, which are annually worth more than 7 billion dollars (Krantzberg and De Boer, 2008; GLFC, 2011). The use of lampricides such as TFM remains an integral component of the sea lamprey control program (Siefkes, 2017; Wilkie *et al.*, 2019). However, the risks of adverse effects on non-target fishes also need to be considered, especially fishes (and other non-target organisms) that are species at risk and/or of cultural importance. Efforts to restore Great Lake’s lake sturgeon populations are ongoing (Golder and Associates Ltd, 2011; Hayes and Caroffino, 2012; LRBOI, 2017) but it is not known how or if these efforts could be undermined by lampricide applications, particularly TFM, which has been identified as a source of age-0 lake sturgeon mortality (Johnson *et al.*, 1999; Boogaard *et al.*, 2003; O’Connor *et al.*, 2017). Indeed, fisheries personnel have attempted to remove lake sturgeon from some lamprey infested streams prior to the application of lampricides, returning them to the stream once the lampricide treatment is completed (LRBOI, 2017). The success of these measures remains unclear but highlights the need to establish measures to protect juvenile lake sturgeon from the adverse effects of TFM. Reducing the concentrations of TFM applied to streams containing YOY lake sturgeon has previously been tried, but this resulted in increased numbers of residual lampreys that survived treatment, ultimately leading to increased parasitic juvenile lamprey populations and greater damage to fisheries (Dobiesz *et al.*, 2018).

Not treating streams containing lake sturgeon is unfeasible because this too would lead to increased numbers of parasitic sea lamprey. In fact, lake sturgeon themselves would be more vulnerable to predation because they too are a target of parasitic sea lamprey (Patrick *et al.*, 2009). Using generalized linear models, Dobiesz *et al.* (2018) recently demonstrated that the benefits of protecting age-0 lake sturgeon by suspending TFM treatments of their nursery streams would be greatly outweighed by the increased mortality of sub-adults (age, 7–24) due to increased sea lamprey attacks. Although suspending lampricide applications to systems containing sturgeon streams would increase adult sturgeon populations by 5.7% in the Great Lakes, they demonstrated that increased sea lamprey parasitism of sub-adults could ultimately lead to reductions in adult lake sturgeon populations of up to 37% (Dobiesz *et al.*, 2018).

Recently, Hepditch *et al.* (2019) demonstrated that the greater susceptibility of lake sturgeon to TFM toxicity in their early juvenile stages (<100 mm in length) resulted from substantially higher mass-specific rates of TFM uptake compared to larger 1+ animals. For this reason, delaying TFM treatments until the autumn when the fish are larger, the rates of TFM uptake much lower, and their capacity to detoxify the lampricide expected to be higher, would be a suitable mitigation measure. Disturbances to ATP production, liver glycogen reserves and glucose supply to the central nervous system (CNS) would also likely be less when the animals were larger, improving the likelihood of post-exposure survival by allowing the sturgeon to restore these energy reserves more quickly. Disturbances to muscle glycogen reserves would also be less, leading to less potential impairment of the fish’s burst and prolonged swimming capacity, improving their ability to evade predators or other threats during or following TFM exposure. Consideration might also be given to applying TFM at lower concentrations but over longer periods (e.g. 18–24 h rather than 12 h), which could result in less TFM accumulation by the sturgeon, while reducing the magnitude of the metabolic disturbances they experience. This would be dependent, however, on the lake sturgeon having a greater capacity to detoxify TFM compared to sea lamprey, which remains to be determined.

Clearly, more work needs to be done to reconcile lake sturgeon conservation and restoration efforts with sea lamprey control efforts. As the present study and our earlier work (Hepditch *et al.*, 2019) reveals, one way to find solutions to the twin challenges of invasive species control and conservation is to learn as much as we can about how interventions (i.e. lampricides) affect not only the physiology of the target organism(s) such as the sea lamprey, but also susceptible non-target organisms such as the lake sturgeon.

## Funding

This work was funded by a research contract awarded by the Great Lakes Fishery Commission (project ID 2016_WIL_54050) to M.P.W (co-applicants L. O’Connor, O. Birceanu and J. M. Wilson).
